# Bioconversion of Lignocellulosic Biomass into Value Added Products under Anaerobic Conditions: Insight into Proteomic Studies

**DOI:** 10.3390/ijms222212249

**Published:** 2021-11-12

**Authors:** Martha Inés Vélez-Mercado, Alicia Guadalupe Talavera-Caro, Karla María Escobedo-Uribe, Salvador Sánchez-Muñoz, Miriam Paulina Luévanos-Escareño, Fernando Hernández-Terán, Alejandra Alvarado, Nagamani Balagurusamy

**Affiliations:** 1 Laboratorio de Biorremediación, Facultad de Ciencias Biológicas, Ciudad Universitaria de la Universidad Autónoma de Coahuila, Carretera Torreón-Matamoros km. 7.5, Torreón CP. 27276, Mexico; marthavelez@uadec.edu.mx (M.I.V.-M.); alicia_talavera@uadec.edu.mx (A.G.T.-C.); karla.uribe@uadec.edu.mx (K.M.E.-U.); miriam_luevanos@uadec.edu.mx (M.P.L.-E.); fernandohernandezteran@uadec.edu.mx (F.H.-T.); 2 Bioprocesses and Sustainable Products Laboratory, Department of Biotechnology, Engineering School of Lorena, University of São Paulo (EEL-USP), Lorena 12602-810, SP, Brazil; salvador.sanchez@usp.br; 3 Interfaculty Institute for Microbiology and Infection Medicine Tübingen, University of Tübingen, Auf der Morgenstelle 24, 72076 Tübingen, Germany

**Keywords:** lignocellulose substrates, biofuels, value added products, anaerobic conditions, proteomics

## Abstract

Production of biofuels and other value-added products from lignocellulose breakdown requires the coordinated metabolic activity of varied microorganisms. The increasing global demand for biofuels encourages the development and optimization of production strategies. Optimization in turn requires a thorough understanding of the microbial mechanisms and metabolic pathways behind the formation of each product of interest. Hydrolysis of lignocellulosic biomass is a bottleneck in its industrial use and often affects yield efficiency. The accessibility of the biomass to the microorganisms is the key to the release of sugars that are then taken up as substrates and subsequently transformed into the desired products. While the effects of different metabolic intermediates in the overall production of biofuel and other relevant products have been studied, the role of proteins and their activity under anaerobic conditions has not been widely explored. Shifts in enzyme production may inform the state of the microorganisms involved; thus, acquiring insights into the protein production and enzyme activity could be an effective resource to optimize production strategies. The application of proteomic analysis is currently a promising strategy in this area. This review deals on the aspects of enzymes and proteomics of bioprocesses of biofuels production using lignocellulosic biomass as substrate.

## 1. Introduction

Biofuels are gaining attention due to the environmental concerns caused by the increasing emissions of greenhouse gases. Although biofuels are practical alternatives to replace fossil fuels, edible sources (soybean, rapeseed, etc.) are frequently used as substrates, which could limit the possibility of meeting the growing energy demand [1]. Hence, alternative biofuels from non-edible substrates have garnered increased attention [2]. Plant-derived biomass (i.e., lignocellulose) is the most abundant sustainable source and promising feedstock to produce biofuels (e.g., bioethanol, biobutanol, and biodiesel) and other value-added products (e.g., biomaterials and biochemicals) [3,4].

In general, aerobic bioconversion of lignocellulolytic substrates and their microbiology have been extensively studied. Mostly, fungi are employed due to their extracellular cellulases, xylanases and ligninase enzymes and their activity potential [5]. Additionally, their mechanisms of degradation involve less steps. Since lignin degradation is mediated by the incorporation of oxygen atom to facilitate cleavage of the aromatic ring, various strategies such as bioreactors design, increased oxygen transfer mechanisms, etc. have been implemented to improve the efficiency of the bioconversion process [6,7].

However, anaerobic breakdown of lignocellulose requires a consortium of several microorganisms, where lignin degradation and cellulose hydrolysis are important rate-limiting steps. Anaerobic cellulolytic microorganisms employ cellulosome, a protein complex that allows binding to the raw substrates and enhances hydrolysis of polysaccharides (more in Section 3.1.). Cellulosome activity is well studied in many *Clostridium* bacteria. A notable example is the strain *C. thermocellum*, which has been deemed a promising candidate for biotechnological applications [8]. Earlier, Shinoda et al. [9] compared two different strains of cellulosome-producing clostridia, viz., *C. thermocellum* and *C. clariflavum*, and concluded that *C. thermocellum* showed a cellulolytic activity of 4.1 U/mg with phosphoric acid swollen cellulose and 0.35 U/mg with avicel, while *C. clariflavum* recorded 2.6 and 0.16 U/mg, respectively. Conversely, they reported that *C. clariflavum* demonstrated higher hemicellulolytic activity of 2.4 U/mg with xyloglucan and 1.7 U/mg with mannan, and *C. thermocellum* registered 1.6 and 1.3 U/mg, respectively. Moreover, recent work where *C. thermocellum* expressed β-glucosidase from a heterologous system, indicated that collective activity of cellulosome enzymes and β-glucosidases positively increases cellulose hydrolysis by this bacterium [10]. Additionally, *C. thermocellum* have the metabolic pathway to produce ethanol as a one-step process directly from cellulose, and a maximum theoretical ethanol yield of 75% can be obtained [7]. *C. thermocellum* have been reported in anaerobic digesters as well and have been related with increases in methane production [11].

As mentioned previously, one of the major bottlenecks is due to the complex structure and recalcitrance of lignin [12], and many authors suggest that pretreatment could aid in removal of 20–60% lignin fraction, which is dependent on solids content, enzyme activity, etc. A yield of about 60-80% sugar has been reported after the pretreatment steps [13,14,15]. The efficiency and yield in the bioconversion of lignocellulosic biomass into biofuels and other value-added processes under anaerobic conditions depend on the development of yield efficient and cost-effective lignin removal processes.

Multi-omics analyses, *viz*., combination of metagenomic, proteomics, transcriptomics, metabolomics offer important tools in deciphering the microbial diversity, identification of key proteins and designing of suitable microbial consortia for production of biofuels and other value added bioproducts. In particular, proteome provides the measurement of expression and activity state of proteins in a cell [16,17]. Through proteomics, data on the structural and functional elements present in the cell and as well as their molecular interactions in biological processes are obtained [18]. In the context of biofuel production, proteomics has been integrated to complement the understanding and regulation of cellular processes, to identify biomarkers for monitoring and to evaluate scaling-up options [19,20,21]. Further, proteomics studies are mainly focused on identifying proteins associated with plant polysaccharide depolymerization [22,23], stress tolerance and metabolic responses to varied treatments [24,25,26]. Additionally, genomic technologies facilitate the design and modification of microbial strains to obtain increased efficiency and yield [27,28,29].

This review focuses on role of different proteins and proteomic insights on the anaerobic bioconversion of lignocellulose substrates for biofuel production and other value-added products.

## 2. Importance of Proteomic Technologies in Bioprocesses

The use of lignocellulosic feedstocks for production of biofuels and other chemicals has gained strength over time. The characterization of lignocellulose and a detailed understanding of its degradation process is critical. Some of the key proteins that have been identified in lignocellulose degradation are cellulases [30], xylanases [31], peroxidases and laccases [32], as well as glycoside hydrolases or GHs [33,34]. Transcriptomics, proteomics, chemoproteomics and metabolomics are used to map, measure or sequence biomolecules from microbial communities. These technologies aid in gathering information related to novel genes, gene functionality, genomic structure, metabolic pathways, and the evolutionary history of the microorganisms implicated in biofuel production. In brief, genomic and transcriptomic technologies help in understanding the genetic elements and their regulation (DNA & RNA), while proteomics provides information on the structural and functional characterization of protein products [17,35]. The obtained molecular information contributes to the development of novel strategies to recover resources and energy from recalcitrant substrates to meet the biofuel demands of the future generations [3].

**Figure 1:**
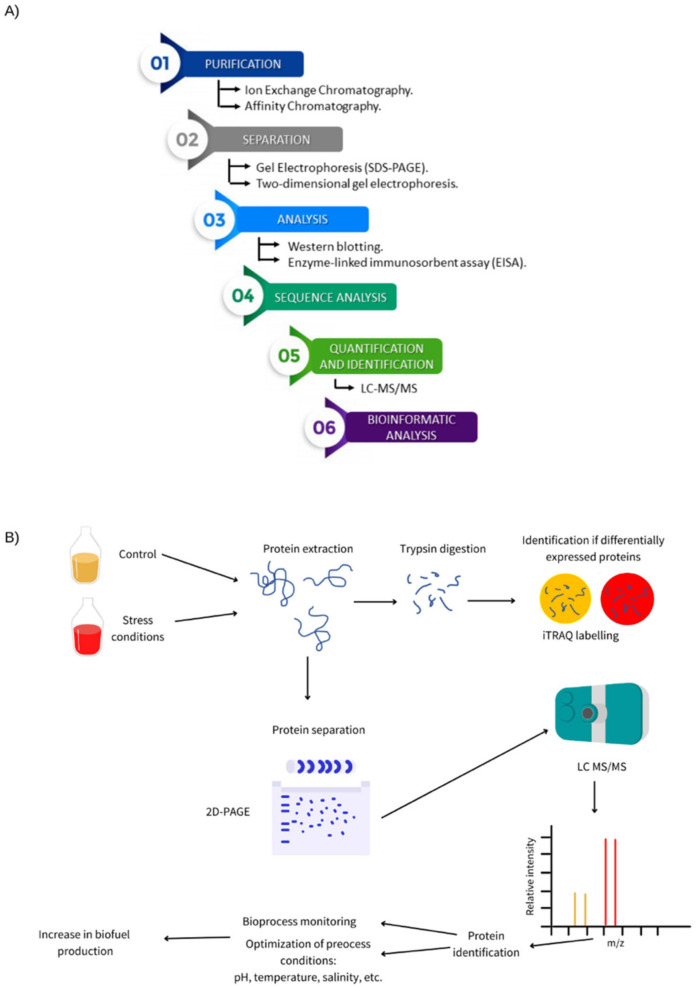
(**A**) Schematic outline for the identification of proteins. (**B**) Optimization and monitoring of bioprocess through the identification of functional proteins.

Some proteomic samples tend to be complex and have an abundance of different components, which makes gel-based techniques (2D PAGE) unsuitable for their analysis. However, there are other methods that can be used for high-throughput proteomics, such as LC-MS/ MS, which generally has two different approaches differentiated by upstream sample preparation methods. The most common approach is bottom-up proteomics, where the protein samples are digested prior to the LC-MS/ MS analysis (1D, 2D and Multidimensional LC can be used). Top-down proteomics is another suitable alternative in which proteins are not digested and are directly analyzed by LC-MS/ MS [36] (Figure 1A). In addition, isobaric tags for relative, and absolute quantitation (iTRAQ) can be used for comparative proteomics to identify the different relative intensity proteins associated with stress conditions and/ or increased biofuel yield [37]. Together, these approaches facilitate the identification of key proteins and their production levels during lignocellulose degradation and fermentation (Figure 1B).

## 3. Proteins Involved in Lignocellulose Utilization

Lignocellulosic biomass is mainly composed of 40–60% cellulose, 20–40% hemicellulose, and 10–24% lignin, but in general, composition varies in different cell walls depending on the plant species [38]. Different degradative enzymes are implicated in the breakdown of the lignocellulosic structure and the hydrolysis or oxidation of the polymers present in plant-derived biomass [39].

**Figure 2:**
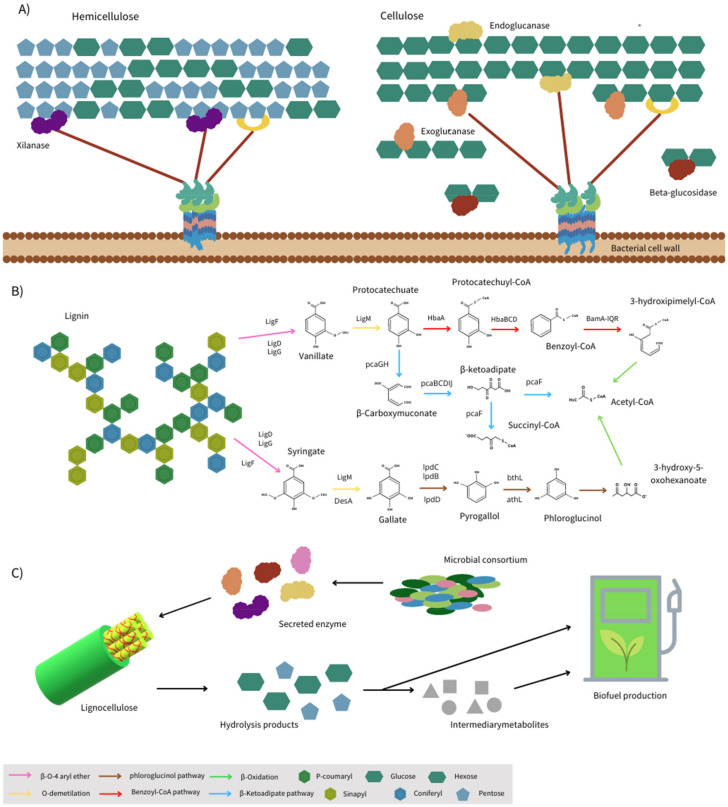
(**A**) Enzymatic mechanisms of the degradation of polysaccharides, (**B**) lignin degradation and (**C**) its potential for the production of by-products.

The recalcitrant nature of lignocellulose [40] makes its hydrolysis a limiting step, and thus converting lignocellulose into biofuel could be an expensive process [41]. Thus, facilitating hydrolysis and subsequent polysaccharide conversion is critical to improve biofuels production feasibility and competitivity against other fuel alternatives. Most of the proteomics studies on the degradation of lignocellulosic biomass to obtain value-added products has been primarily employed to determine hydrolytic enzymes, particularly, the extracellular enzymes secreted by microorganisms (Figure 2C), which together are known as the secretome [42]. The simple sugars obtained after the hydrolysis of polysaccharides by secretome enzymes are readily utilized as a carbon source for biofuel [43]. Different enzymes that participate in hydrolysis of lignocellulosic substrates and their mechanisms are presented in this section.

### 3.1. Enzymes Targeting Lignocellulosic Polysaccharides

Cellulose, one of the major components of lignocellulose, is a homopolysaccharide with amorphous and crystalline regions [44] made of glucose monomers linked by β-1,4-D-glucan. Hemicellulose however is a heterogenous polysaccharide conformed by a xylan backbone that contains xylose, arabinose, mannose, glucose, galactose and sugar acids in different proportions depending on the source [43,45].

Hydrolytic enzymes capable of acting on cellulose and hemicellulose are called cellulolytic and hemicellulolytic enzymes, respectively, and belong to glycoside hydrolases (GH), which are grouped under CAZy (Carbohydrate-Active enZymes). GHs are classified according to its primary sequence into 168 families in the CAZy database [46]. A given GH enzyme may belong to a particular family based on its specific characteristics, such as protein structure, enzymatic activity, specificity, and reaction mechanism [47]. In general, two different mechanisms, *viz*., inversion and retention, are employed by GH families to cleave glycosidic bonds [48,49].

Three cellulolytic enzymes (exoglucanase, endoglucanase, β-glucosidase) play a major role in cellulose biodegradation. Exoglucanases (EC 3.2.1.91) as well as endoglucanase (EC 3.2.1.4) employ either inverting or retaining mechanisms to hydrolyze the β-1,4 linkages of cellulose in amorphous and crystalline regions, respectively. Meanwhile, β-glucosidase (EC 3.2.1.21) act synergistically with exoglucanases and endoglucanases, by hydrolyzing the β-1,4 linkages of a cellulose-derived disaccharide (i.e., cellobiose) [50,51]. The major enzymes involved in cellulose hydrolysis expressed by a variety of microorganisms; especially, *Clostridium* genera are described in Table 1.

A greater number of enzymes are involved in the degradation of hemicellulose due to its heterogenous structure. In addition to GH, hemicellulases include carbohydrate esterases (CEs). Since xylan is the major component of hemicellulose, xylanases (EC 3.2.1.8) are one of the main enzymes involved in hemicellulose depolymerization by cleavage of the β-1,4 linkages of the xylan backbone, producing xylooligomers such as xylobiose and xylose. Besides xylanases, β-Xylosidases, α -L-arabinofuranosidases, β-mannanases, β -mannosidases and α-glucuronidases also play an important role in the breakdown of hemicellulose and have been identified by proteomic analyses by several authors (Table 1 and Table 2). It can be seen from Table 1 and Table 2 that there are multiple reports on anaerobic hydrolysis of cellulosic biomass. However, in the case of lignin degradation, the available reports are on aerobic bioconversion of lignin.

β-xylosidases (EC 3.2.1.37) act upon the β -1,4 bonds on the nonreducing ends of xylooligomers, xylobiose and, in some cases, on xylan by employing a retaining mechanism. Meanwhile, α-L-arabinofuranosidases (EC 3.2.1.55) are arabinases that hydrolyze α-L-arabinofuranosyl groups acting on α-L-1,3 and α-L-1,5 linkages of arabinans, arabinoxylans and arabinogalactans. Enzymes α-L-arabinofuranosidases use retaining or inverting mechanisms. Meanwhile, β-mannanases (EC 3.2.1.78) and β -mannosidases (EC 3.2.1.25) act sequentially, β-mannanases hydrolyze mannan-based saccharides to produce β-1,4-manno-oligomers, which are then hydrolyzed by β-mannosidases to yield mannose. Finally, α-glucuronidases (EC 3.2.1.131) [43,63] hydrolyze α-1,2 linkages between xylose and D-glucuronic acid by inverting mechanism. *Clostridium* spp. encode most of these hemicellulolytic enzymes (Table 1) and has shown a great potential for genetic engineering to improve lignocellulose hydrolysis.

Hemicellulolytic carbohydrate esterases remove the ester group from carbohydrates and facilitates the access to GHs [64]. Esterases are classified into 18 families according to the CAZy database (www.CAZy.org; accessed on 23 September 2021), these include feruloyl esterases (EC 3.1.1.73) from the CE1 family which catalyze the cleavage of the ester bond at the O-5 position between a ferulic acid and arabinose liberating hydroxycinnamic acids; as well as acetyl xylan esterases (EC 3.1.1.72), which in turn catalyzes the cleavage of ester linkages on the position O-2 and O-3 between an acetyl group and xylose [45,65]. Various studies reported higher expression of hemicellulases than cellulases, as hemicellulose is more exposed than cellulose [52,53]. In general, the polysaccharides are hydrolyzed either by extracellular enzymes and/ or by the cellulosome (Figure 2A).

### 3.2. Enzymes Involved in Lignin Degradation

Lignin is one of the most complex substrates compared to the other components of the lignocellulosic structure (cellulose and hemicellulose), and its depolymerization involves a variety of enzymes (Table 3). Under aerobic conditions, the main degradative enzymes are peroxidases and laccases, which need molecular oxygen for their catalytic activity [66]. On the contrary, several enzymes participate in lignin degradation under anaerobic conditions. Auxiliary Activities (AAs) are a recent family of catalytic proteins in the CAZy database, which are redox enzymes and are classified into 16 subfamilies, including different peroxidases (like lignin peroxidase and manganese peroxidase) and laccases, which are known to act on lignin. Other enzymes in this classification include oxidases, demethylases, and reductases [67].

Lignin is an aromatic heteropolymer of phenylpropanes (mainly coniferyl, p-coumaryl, and sinapyl alcohols) bound to hemicellulose and cellulose and intermolecularly connected by carbon–carbon and aryl–ether linkages [40,73]. β-O-4 aryl ether bonds are the most predominant intermolecular bonds present on lignin which represent the 45–60% of the total linkages [69]. Three main enzymes, *viz*., Cα-dehydrogenase, β-etherase and glutathione lyase are implicated in the breakdown of the β-O-4 aryl ether bonds. Cα-dehydrogenase is a NAD^+^ dependent enzyme that oxidizes benzyl alcohol at Cα position increasing the polarity, which facilitates the β-etherase activity. The glutathione dependent β-etherase breaks the ether bond by the addition of glutathione that is later eliminated by glutathione lyases [68]. These three intracellular enzymes cannot act on high molecular weight lignin. Otsuka et al. [80] reported a β-etherase that does not need glutathione and uses molecules of water to cleavage at Cα and Cβ positions of the β-O-4 aryl ether bonds extracellularly [81]. Lignin depolymerization results in different lignin derivatives, and the most common are vainillate and syringate. Subsequently, demethylation of vainillate and syringate by vainillate O-demethylase and syringate O-demethylase, respectively, are important steps to produce protocatechuate and gallate, which as intermediaries enter different pathways for ring cleavage [70,82]. Under anaerobic conditions, protocatechuate and gallate are converted to different key intermediates such as benzoyl-CoA, phloroglucinol, hydroxyhydroquinone and resorcinol [73]. Benzoyl-CoA, being the most common intermediate, is used as biomarker in anaerobic degradation of aromatic compounds [83]. A schematic diagram of the main reactions involved in anaerobic lignin degradation, key intermediates and ring cleavage is shown in Figure 2B. Protocatechuate anaerobic degradation can be via the benzoyl-CoA pathway or β-ketoadipate pathway, whereas gallate anaerobic degradation takes place by the phloroglucinol pathway [82]. The benzoyl-CoA pathway consists of four main steps, which are (i) activation, (ii) ring reduction, (iii) ring cleavage and (iv) conversion to acetyl-CoA [71,74]. The participating enzymes of this pathway are summarized in Table 3. The β-ketoadipate pathway is a conserved metabolic route that starts with the protocatechuate ring cleavage by protocatechuate 3,4-dioxygenase resulting in β-carboxymuconate, which passes through several reactions to form β-ketoadipate, which is then ligated to a coenzyme A by a transferase and finally separated into succinyl-CoA and acetyl-CoA [84].

Phloroglucinol is an intermediate found during gallate anaerobic degradation. Gallate initially undergoes decarboxylation by gallate decarboxylase forming pyrogallol, which is then converted to phloroglucinol by the transfer of a hydroxyl group. Then the phloroglucinol ring is cleaved by a hydrolase and the resulting product undergoes β-oxidation to obtain acetyl-CoA [78,79]. Table 3 enlists important enzymes of different pathways that target lignin or its derivatives in anaerobic conditions; the genes that encode these enzymes are also indicated. Although there are no complete proteomic analyzes on anaerobic ligninolytic enzymes, genomic and transcriptomic analyses have been employed for their identification [82,85,86].

## 4. Biofuel Production from Lignocellulosic Biomass

The interest in the production of biofuels from renewable sources has increased in the recent years due to environmental concerns and the concomitant need to decrease our dependence on fossil-based energy resources [1]. Among the potential substrates, lignocellulose is a major renewable source with potential for application in various bioprocesses for production of value-added products [87]. Microbial production of different biofuels such as ethanol, methane, hydrogen, butanol and others using lignocellulosic residues as carbon source is presented in Table 4.

Different strategies such as chemical pretreatments or/and enzymatic hydrolysis have been employed to recover the energy from lignocellulosic biomass (Table 4). However, biofuel yield varies widely depending on the choice of pretreatment and the microorganism employed. In general, chemical and mixed pretreatments achieved significant hydrolysis of hemicellulose and soluble lignin (Table 4). However, the formation of various inhibitory substances during chemical pretreatments limits recovery and yield [99]. Conversely, enzymatic hydrolysis is gaining attention to overcome inhibitory substances and thus improve yield. More information at the molecular level is needed to devise novel strategies to increase biofuel recovery. In this regard, proteomic analysis may reveal which enzymes are missing/present at distinct steps of treatment, thereby permitting a snapshot of the microbial activity, their metabolism and protein production. This in turn could favor the identification of biomarkers for optimizing and monitoring of the bioprocess.

### 4.1. Proteomics of Ethanol Production

Lignocellulosic biomass has been widely used for industrial production of bioethanol [100,101]. In general, the lignocellulosic biomass undergoes pretreatment for liberation of sugars, which are fermented to bioethanol. The interaction between cellulose hydrolytic enzymes is necessary for cellulose hydrolysis before the fermentation process starts. Based on the proteome analyses of several studies, there are some key proteins during ethanol production (Table 5). For example, alcohol dehydrogenase, acetaldehyde-CoA/alcohol dehydrogenase, pyruvate formate lyase and glyceraldehyde-3-phosphate dehydrogenase.

According to Usai et al. [22], the cellulolytic bacterium *Clostridium cellulovorans* showed different kinetics and energetics based on the substrate that is used. This study showed global changes in *C. cellulovorans* proteome when grown on crystalline cellulose (avicel) and a soluble carbohydrate (glucose). Notably, ATP-dependent 6-phosphofructokinase, the principal regulatory enzyme for glycolysis pathway was upregulated when *C. cellulovorans* grew on avicel [106].

Moreover, Usai et al. [22] identified pyruvate phosphate dikinase (PPDK) as a putative key enzyme in the regulation of carbon flux during cellulose metabolism. They also reported that phosphoenolpyruvate carboxylase (PEPC), found in similar amounts in avicel and glucose, could replace the phosphoenolpyruvate carboxykinase (PEPCK) activity in the malate shunt, an alternative pathway for the conversion of phosphoenolpyruvate (PEP) to oxaloacetate. In addition, few alcohol dehydrogenases were upregulated in avicel, of which Clocel_3817 (an iron-containing alcohol dehydrogenase), was the most highly produced. The authors concluded that Clocel_3817 was possibly involved in the reduction of acetyl-coA to acetaldehyde initially, and later to ethanol, concomitantly oxidizing two NADH to NAD. Conversely, a malic enzyme and a glyceraldehyde-3-phosphate dehydrogenase (GAPDH) were downregulated in the presence of avicel. On the contrary, GAPDH, an enzyme associated with bottlenecks in glycolysis pathway, showed upregulation in *C. cellulovorans* with glucose as substrate [22]. There were also three upregulated enzymes, glutamate dehydrogenase, glutamine synthetase and glutamate synthase, which are involved in nitrogen assimilation and synthesis of components of cell biomass. The activity of these enzymes plays a critical role in nitrogen assimilation and are present in most bacterial species.

Poudel et al. [105] reported the proteome of *Caldicellulosiruptor bescii* DSM6725. They analyzed the production of extracellular proteins across C5 (xylose and xylan) and C6 (glucose, cellobiose, avicel) substrate classes. Extracellular solute binding proteins (ESBPs) (enzymes that show response to a specific type of substrate) have non-catalytic extracellular activities and are important for lignocellulose deconstruction. Some ESBPs were found to be upregulated with C5 substrates such as the extracellular solute binding protein Athe_0089, an endo-1,4-beta-xylanase which was specific to xylan.

Other extracellular binding proteins (Athe_0523 and Athe_2091), specific to xylose and xylan and related to the hydrolysis of O-glycosyl compounds, were upregulated as well. Unlike C5 substrates, no extracellular solute binding proteins showed upregulation with C6 substrates. The study only recorded the activity of enzymes involved in the synthesis and breakdown of complex polysaccharides [30]. A pair of CAZymes belonging to the GH family were more abundant with avicel than cellobiose alone, despite their cellobiose/cellodextrin phosphorylase activities. Some other enzymes, such as xylose isomerase and ABC transporter-related proteins, were also upregulated with C6 substrates, indicating the importance of glucose transport. ABC transporters are a group of proteins found in the membrane that transport solute molecules via the consumption of ATP [107]. Recently Zurawski et al. [108] reported that these transporters in coordination with CAZymes play an important role in enhancing the usage of the carbohydrate content of plant biomass by *Caldicellulosiruptor* species.

While substrate differences could affect production of enzymes, other parameters that such as presence of other molecules also affect enzymes. For instance, exogenous ethanol or acetic acid addition. Microbial ethanol stress response has generally been described to be a complex biological process. The molecular response to ethanol stress of *Ethanoligenens harbinense* strain YUAN-3, an anaerobic bacterium capable of producing ethanol, acetic acid, hydrogen and CO_2_ was evaluated earlier [102]. They studied the protein production under different ethanol concentrations and reported that the bifunctional acetaldehyde-CoA/alcohol dehydrogenase (ADHE) which generates ethanol from acetyl-CoA plays a key role in ethanol production [109], and showed upregulation at all tested concentrations of ethanol. They observed that ADHE production level was closely related to the endogenous ethanol yield, indicating that ethanol yield increases when exogenous ethanol is added to the medium. In addition, glycolysis related enzymes such as glyceraldehyde-3-phosphate dehydrogenase showed an upregulation at 100 mM ethanol, demonstrating that this condition increases the demand for energy to increase tolerance. Some enzymes involved in ethanol-tolerance stress were also upregulated, for instance, desulfoferrodoxin and glutathione peroxidase, which protect organisms from oxidative stress. Urea carboxylase, allophanate hydrolase and two urea carboxylase-associated proteins were also upregulated during nitrogen metabolism at stress conditions of 50 mM ethanol.

Likewise, acetic acid stress response was evaluated in *E. harbinense* YUAN-3, and upregulation of glyceraldehyde-3-phosphate dehydrogenase (ADU27040) was observed in the presence of 200 mM acetic acid. This enzyme was recently related with the efficient repair of cytotoxic DNA lesions in *E. coli*, and Li et al. [103] suggested its possible role as a response to maintain DNA structure during acetic acid stress.

Phenolic compounds are the main inhibitor of acetone-butanol-ethanol fermentation in Clostridia. In a study by Raut et al. [104], the effect of lignin on cellobiose consumption by *Clostridium acetobutylicum* ATCC 824 was evaluated. Glycolysis, fermentation and associated pathways were significantly repressed in the presence of lignin, this was seen by the downregulation of some enzymes related to solvent production such as acetaldehyde dehydrogenase (CA_C0162) and an aldehyde/alcohol dehydrogenase (Adhe2), in which production has been suggested to be sensitive to culture conditions [110].

### 4.2. Proteomics in the Production of Acids and Solvents

**Figure 3:**
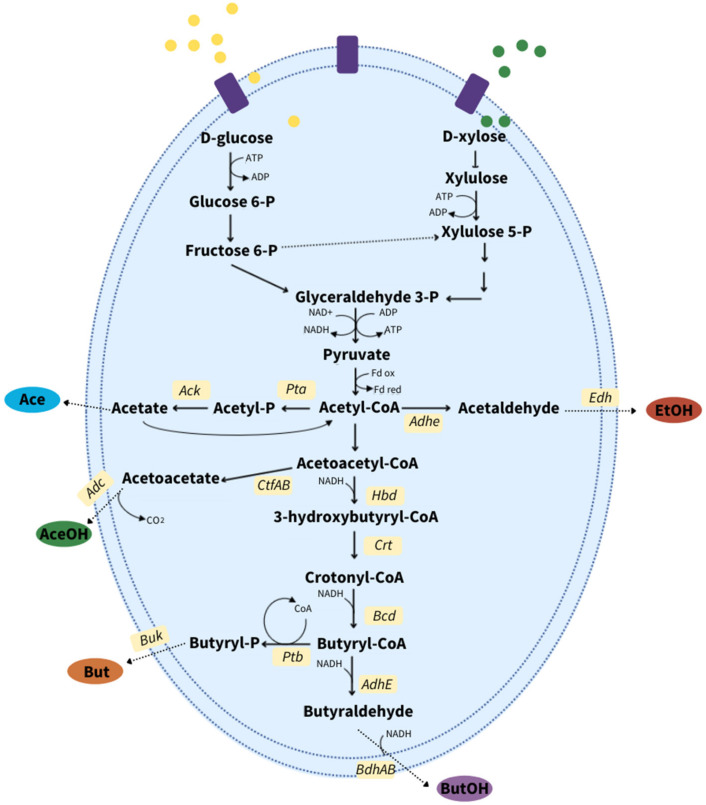
General metabolic pathway studied for value-added products on acidogenic and solventogenic phase performance. Ack: acetate kinase; Pta: phosphotransacetylase; Edh: ethanol dehydrogenase; Hbd: 3-hydroxybutyryl-CoA dehydrogenase; CtfAb: CoA transferase; Adc: acetoacetate decarboxylase; Crt: crotonase; Bcd: butyryl-CoA dehydrogenase; Ptb: phosphotransbutyrylase; Buk: butyrate kinase; AdhE: butyraldehyde dehydrogenase; BdhAB: butanol dehydrogenase; Ace: acetate; AceOH: acetone; EtOH: ethanol; But: butyrate; ButOH: butanol.

Acid–solvent biosynthesis takes place through central carbon metabolism from different sources (glucose and xylose), and their key modulations (redox and energy generation) are well studied in the *Clostridium* genus [111]. When the substrates are metabolized through the central carbon pathways (glycolysis or pentose-phosphate) under anaerobic conditions, microorganisms produce acids from the main intermediary acetyl-CoA (Figure 3). Glyceraldehyde-3-phosphate dehydrogenase is the key enzyme as it generates NADH via glyceraldehyde-3-phosphate oxidation. This has been identified as a bottleneck of sugar metabolism for efficient acid–solvent production [22].

Anaerobes mostly oxidize pyruvate to acetyl-CoA via pyruvate ferredoxin oxidoreductase, which cleaves the carbon-carbon bonds for electron transfer coupling flavoproteins in the reduction of crotonyl-CoA to butyryl-CoA [112]. Acid formation begins from the CoA precursors (acetyl-CoA, crotonyl-CoA), which act to activate expression of genes that produce different enzymes of an organized operon. The activation of this operon for acid formation will depend on the environmental conditions (mainly pH) and the energy requirements from the organism. The phosphate acetyltransferase (pta) and acetate kinase (ack) are strongly related to acid formation as the first step. Moreover, high levels of thiolase A (thlA), crotonase (crt,) and butyryl-CoA dehydrogenase (bcd) were reported to be mainly involved in acetyl-CoA to butyryl-CoA conversion. However, earlier studies indicated that the proteins CAP0036 and CAP0037 in *Clostridium acetobutylicum*, also regulate metabolism under acidogenic conditions [113,114]. Furthermore, under stressful acidic conditions (pH 4.5), fermentation products such as acetate and butyrate and high levels of cofactors such as ATP, NAD(P)H/NAD(P)^+^ serve as signals that trigger a rapid shift in the metabolic pathways from acidogenesis to solventogenesis [115]. Other environmental conditions (temperature, digestion time) along with several stress-inducing compounds such as butyryl-phosphate and formic acid, may also alter cellular activities causing a shift of metabolism from acids production to solventogenesis.

This shift is controlled principally by three enzymes, acetoacetate decarboxylase (Adc), aldehyde/alcohol dehydrogenase (AdhE) and the acetoacetyl-CoA:acyl CoA transferase (CtfA/B), which at the start of the process, are downregulated during acid production but highly expressed during the production of solvents. During this shift, CoA-transferase (CtfA/B) plays an important role in the regulation of the bioconversion of acid precursors into solvents. The presence of the CtfA/B enzyme is consistently linked to solventogenesis (acetone and butanol), since it was observed that its downregulation lowers acetone–butanol production [116,117].

The metabolism switching from acid to solvent production has been analyzed as a strategy developed by microorganisms to alternate the intracellular stress. Despite its importance, solventogenesis still lacks fundamental understanding. Identification of mechanisms that regulate this process is important to increase production without bacterial population decay [118].

Furthermore, solvent butanol–acetone yield is well known to be coupled to pH decrease. At this point, the activity of CoA transferase (CtfAB) and aldehyde/alcohol dehydrogenase (AdhE1) increases, which induces the solventogenesis and transfer of electrons through flavodoxins, ferredoxin and thioredoxin [119]. The H_2_-uptake hydrogenases regulate the flow of electrons and are actively expressed during acidogenesis. Additionally, Nakayama et al. [120] indicated that energy transfer has a key role in solventogenesis and reported that hydrogenases were upregulated to increase acetone/butanol yield.

In another study, alcohol dehydrogenase (ADH) production was observed in *Acinetobacter* strains during ethanol conversion to acetate, the primary pathway of reversion of solventogenesis. The ADH has been related to bacterial quorum sensing, and as a key stimulator for alcohol oxidation [121]. Conversely, spore formation has been reported as a survival mechanism under solventogenesis stress. Spo0A has been identified as a global regulator of solvent production. The overexpression of this gene, *spo0A*, in *C. acetobutylicum* resulted in the upregulation of acetoacetate decarboxylase (CAP0165) and butanol dehydrogenase (CAC3299), while acetate kinase (CAC1743) and butyrate kinase (CAC2075/CAC1660) were downregulated [122].

### 4.3. Proteomics of Methane and Hydrogen Production

Production of biohydrogen and methane is undertaken by specific groups of microorganisms. Microorganisms from the genera *Halothermothrix*, *Syntrophomonas* and *Clostridium* are important players in the production of hydrogen [123]. Further, acetate accumulated during the processes is oxidized by syntrophic bacteria into H_2_ and CO_2_. Methanogenesis is a slow reaction and sensitive to inhibitory factors (e.g., ammonium and sulfide) [124]. Many studies have identified several enzymes associated with aceticlastic and hydrogenotrophic-methanogenesis pathway, *viz*., F_420_ non-reducing hydrogenase/heterodisulfide reductase complex, methyl-coenzymeM reductase, tetrahydromethanopterin S-methyltransferase [123,125]. High abundance of enzymes involved in methanogenesis, either the key enzyme of hydrogenotrophs (5,10-methylenetetrahydromethanopterin reductase) or the acetoclastic methanogens (acetyl-CoA decarbonylase/synthase) are essential [126,127]. The most important step involved is the production and activity of the key enzyme, methyl coenzyme M reductase (encode by the gene *mcrA*) to increase methane production [128].

Methane production and organic acids production are reported to be directly correlated to methane percentage and molar values of acids (i.e., acetate) [129]. This fermentation step is correlated to all the further steps of production. NiFe and Fe–Fe hydrogenases are the most common and both use the NAD(P)H as a donor and reduce ferredoxin proteins for hydrogen production [130]. Further, periplasmatically oriented hydrogen-oxidizing and a cytoplasmatically oriented putative H_2_-producing membrane bound hydrogenases have been reported in *Sulfospirillum multivorans* [131].

The distinct protein production profile during biohydrogen and methanation is influenced by several factors that induce stress to the cell; for example, acid tolerance is one of the limitations that these processes face. A study reported that at 7.5 g/L of butyrate presents a positive stress for protein abundance to overcome the stress and avoid affecting production. Nonetheless, when the concentration raised to 15 g/L, negative stress was observed lowering protein production. Performance of the process in the same study, reported the proton transfer as the main factor under this stress conditions of acidification, were dehydrogenases played a key role. The principal enzymes synthesized under acidification were dehydrogenase and methyltranferases proteins related to methane production [132]. Conversely, for sole H_2_ production the electron-transfer flavoprotein, hydrogenase expression/formation protein (hupG) and phosphate butyryl transferase (sp2) were known for their role in H_2_ production, especially the sp2 is mostly expressed when higher concentrations of butyrate are present, similarly to methane production [133].

Other studies report the influence of temperature on regulation and protein folding, where a decrease in temperature results in the overproduction of heat shock proteins (HSp). In biomethanation, it has been shown that the mechanisms of cells will change the regulation and high expression of genes encoding for nucleic-acid-binding proteins (CspA-related proteins) and chaperones (DnaK and GroEL) [134]. In contrast, at higher temperatures (30–55 °C), Hsp70 and Hsp60 enzyme stress systems are upregulated in order to assist protein folding. Under thermophilic conditions, HSp are thermostability indicators, although it has been found that protein synthesis levels of key enzymes involved in methane metabolism at high temperatures takes place. Hydrogenotrophic enzymes such as acetate kinase and the acetyl-CoA decarbonylase/synthase complex were detected at 55 °C [135,136]. Moreover, HSp are also present when other forms of stress affect the microbial community, such as high ammonium and high salt concentrations [135].

## 5. Conclusions

Although lignocellulose is a highly recalcitrant material, its abundance makes it an ideal candidate to produce biofuels, such as bioethanol and methane, as well as other products of value, such as organic solvents and acids. Hydrolysis of plant biomass components under anaerobic conditions is carried out by microbial consortia and several enzymes need to act synergistically. Members of Firmicutes, Bacteroidetes, Proteobacteria play a major role, where *Clostridium* spp. are the most well-studied cellulolytic bacteria and are also industrially exploited for cellulose catabolism. In general, cellulases and hemicellulases, which are GHs, hydrolyze glycosidic bonds. These GH enzymes are classified into more than one hundred families depending on their protein sequence. Besides GH, other enzymes take part in the breakdown of cellulose including polysaccharide lyases, carbohydrate esterases and auxiliary activities (Table 1). Moreover, the production of methane requires the presence of multiple enzymes, primarily methyl coenzyme M reductase, whose upregulation is associated with increased methane production.

It is well known that the bioconversion of plant biomass requires several pretreatment strategies, including chemical, mechanical and enzymatic treatments or a combination of different methods (Table 4). Chemical pretreatments have been found to yield higher ethanol titers; however, chemical treatments result in the formation of several compounds that inhibit enzymatic activity later in the process. Additionally, several intermediates such as volatile fatty acids and alcohols formed may induce cellular toxicity. Thereby, making the process of biofuel production from plant biomass practical and efficient requires a thorough knowledge of the proteins, primarily the enzymes involved in the conversion of complex sugars into ethanol, methane and other compounds, and of the proteins involved in mechanisms of stress tolerance. In this context, proteomics is a promising technology that can be used to identify proteins of interest that could aid in identification and development of engineered microbial pathways and monitoring strategies. Hence, this review highlights the involvement of the wide variety of enzymatic proteins during the bioprocess of biofuel production from lignocelluosic feedstocks under anaerobic conditions.

Proteomics has been used earlier to characterize the proteins present in plant biomass, and these studies have primarily addressed the composition of plants cell walls from different sources [137,138]. Meanwhile, studies on the microbial proteome tend to focus on one microorganism growing in the presence of a particular polysaccharide. Earlier studies have shown the differences between important microbial enzymes when distinct microorganisms were grown in the presence of varied substrates (Table 5). Although several proteins involved in central and pyruvate metabolism as well as ethanol production have shown differential abundance, patterns among the substrates, conditions and microorganisms involved are imperceptible. Multivariable studies where the same complex inoculum or several bacteria are employed under diverse conditions, may reveal whether certain functional groups of proteins display similar production patterns. However, due to the high complexity behind biofuel production, it is our view that multivariable analyses may not reveal comprehensive principles that could be applied to all bioprocesses. Instead, we postulate that employing proteomic studies will aid to improve the design and application of a specific bioprocess, where distinct proteins could be identified as efficiency biomarkers at every stage of that unique process, or where certain enzymes could be targeted for metabolic engineering to increase production of a desired compound.

In general, previous studies have shown how proteomics could be applied in the context of biofuel production and for process improvement. For instance, quantitative proteomics by tandem mass spectrometry identified unique upregulated proteins corresponding to photosystems of a cyanobacterial species [139], which suggested that these proteins could be targets to design ethanol-tolerant superior strains. Similarly, proteomics analyses of a *Clostridium* strain grown on different carbon sources, including cellulose and hemicellulose, allowed the identification of key enzymes that participate in the breakdown of each distinct substrate, these enzymes could in turn be used as targets to engineer this bacterium to favor the uptake of a particular substrate [140]. In a more recent study using *Clostridium cellulovorans*, it was observed that global proteome profiles were carbon source-dependent, with notable differences in the upregulation of ATP-biosynthesis enzymes in cells grown on cellulose [22]. Meanwhile, a study of *Clostridum acetobutylicum* grown on different polysaccharides, including lignin, identified multiple metabolic pathways and proteins that are repressed in the presence of lignin. These proteins included ATP-dependent cell division factors, which were deemed part of the “lignin bottleneck” for this organism, and could be used as biomarkers to monitor the presence of this *Clostridum* strain or for modification and engineering [104] Moreover, another study focused on two cellulolytic Bacteroidetes strains using label-free protein quantification coupled with cell fractionation revealed proteins present when the strains grew on two distinct polysaccharides and their subcellular localization. The results identified strain-specific enzymes and previously unstudied GHs [141], and these proteins could also be used as biomarkers for these strains.

Thus, proteomics analyses can retrieve direct protein production signatures, such as accumulation or decrease of particular enzymes. These data could help in the identification of target enzymes that could be in turn engineered to avoid metabolic bottlenecks that are encountered in using lignocellulosic feedstocks. Additionally, proteins interact allosterically with multiple molecules, and hence proteomics studies coupled with crosslinking and mass-spectrometry-based identification in the context of bioconversion could be used to map such interactions, and in turn aid in the design of improved microbial strains engineered for optimal activity. At the moment, applications of proteomics are process-specific, but in the future, a combination of multiple biological scales, i.e., proteomics, transcriptomics and metabolomics, may lead to the development of machine learning tools that can predict and design strategies for the bioconversion of recalcitrant feedstocks into biofuels and other value-added products.

## Figures and Tables

**Table 1:** Enzymes involved in depolymerization of different polysaccharide substrates and their microbial source.

	EC Number	Putative Function	Organism Source	Substrate	Activity or Function	References
Cellulose	3.2.1.21	β-glucosidase	*Bacteroides coprosuis* *Roseburia intestinails* - *Clostridium termitidi* - *Pantoea ananatis* Sd-1	Corn stover - α-cellulose and cellobiose - Rice straw	Cleavages β-1,4 linkages of cellobiose	[52] - [53] - [54]
	3.2.1.4	Endo-β-1,4-glucanase -Endoglucanase	*Cellulosilyticum lentocellum* *Clostridium cellobioparum* *Clostridium cellulolyticum* *Eubacterium cellulosolvens* *Clostridium saccharoperbutylacetonicum* - *Clostridium cellulolyticum* *Clostridium josui* *-* *Clostridium termitidi* *-* *Pantoea ananatis* Sd-1	Corn stover - Filter paper - α-cellulose and cellobiose - Rice straw	Hydrolyzes β-1,4 bonds in the amorphous regions of cellulose	[52] - [55] - [53] - [54]
	3.2.1.91	Cellobiohydrolase – Exoglucanase - 1,4- β-cellobiosidase	*Clostridium saccharoperbutylacetonicum* *Clostridium cellulyticum* *Clostridium ruminicola* *-* *Clostridium termitidi* *-* *Clostridium josui* *-* *Pantoea ananatis* Sd-1 *-* *Caldicellulosiruptor bescii* *Caldicellulosiruptor obsidiansis*	Corn stover - α-cellulose and cellobiose - Filter paper - Rice straw - Crystalline cellulose	Attack crystalline cellulose in the β-1,4 linkages	[52] - [53] - [55] - [54] - [56]
	3.2. 1.86	6-phospho-β-glucosidase	*Clostridium butyricum* *Enterococcus casseliflavus*	Corn stover	Cleavage β-1, 4-linked cellobiose 6-phosphate	[52]
	2.4.1.20	Cellobiose phosphorylase	*Clostridium phytofermentans*	Corn stover	Catalyzes the reversible phosphorolysis of cellobiose	[52]
	NA	Cellulase	*Clostridium cellobioparum* *Clostridium lentocellum Clostridium cellulolyticum* *-* *Caldicellulosiruptor bescii*	Filter paper - Crystalline cellulose	Cleavage the β-1,4 linkages in cellulose	[55] - [56]
	3.2.1.1	α-amylase	*Clostridium saccharoperbutylacetonicum* *-* *Caldicellulosiruptor bescii* *Caldicellulosiruptor obsidiansis*	Corn stover - Crystalline cellulose	Hydrolyze the α-1,4-glucosidic bonds in α-glucans	[52] - [56]
	3.2.1.39	Endo-1,3-β-glucanase	*Caldicellulosiruptor obsidiansis*	Crystalline cellulose	Hydrolyzes β-1,3-bonds present in glucans	[56]
Hemicellulose	3.2.1.8	Xylanase-Endoxylanase- Endo-β-1,4-xylanase	*Cellulosilyticum lentocellum* *Roseburia intestinalis* *Ruminococcus* sp. *Cellulosilyticum ruminicola* *Lachnoclostridium phytofermentans* *Butyrivibrio fibrisolvens* *Clostridium cellulosi* - *Clostridium termitidi* - *Caldicellulosiruptor bescii* *Caldicellulosiruptor obsidiansis*	Corn stover - α-cellulose - Crystalline cellulose	Attack β-1,4 bond of the xylan backbone	[52] - [53] - [56]
	3.2.1.23	β-galactosidase	*Clostridium* sp. *-* *Caldicellulosiruptor bescii* *Caldicellulosiruptor obsidiansis*	Corn stover - Crystalline cellulose	Hydrolyze β-1,4-glycosidic linkage present in lactose	[52] - [56]
	3.2.1.89	arabinogalactan endo-1,4-β-galactosidase	*Paenibacillus* sp. *-* *Caldicellulosiruptor obsidiansis*	Corn stover	Hydrolyze β-1,4 linkages in arabinogalactans	[52]
	3.2.1.25	Endo-1,4-β- mannosidase	*Clostridium clariflavum* - *Caldicellulosiruptor bescii* *Caldicellulosiruptor obsidiansis*	Corn stover - Crystalline cellulose	Cleavage the β-1,4-manno-oligomers	[52] - [56]
	3.2.1.131	α-glucuronidase	*Paenibacillus* sp.	Corn stover	Hydrolyze α-1,2-glycosidic linkage between xylose and glucuronic acid	[52]
	3.2.1.31	β-glucuronidase	*Clostridium cellulovorans*	Corn stover	Exohydrolyze β-d-glucuronic acid residues of glycosaminoglycan	[52]
	3.2.1.37	β-xylosidase	*Sphaerochaeta coccoides* *Clostridium saccharoperbutylacetonicum* *Clostridium ruminicola* *Flavobacterium johnsoniae* *Cellulosilyticum ruminicola*	Corn stover	Exohydrolyze β-1,4 linkages of xylans, to removing xylose residues	[52]
	3.2.1.6	Endo -1,3(4)-β-α- Glucanase	*Clostridium perfringens*	Corn stover	Endohydrolysis of β -1,3 or β -1,4 linkages in β-D-glucans	[52]
	3.2.1.78	β-mannanase	*Clostridium clariflavum* *Roseburia intestinalis* *Cellulosilyticum lentocellum* *-* *Clostridium termitidi*	Corn stover α-cellulose	Attack the β-1,4 bond in D-mannan	[52] [53]
	3.2.1.177	α-xylosidase	*Paenibacillus mucilaginosus*	Corn stover	Hydrolyze α-1,6 linked xylose residues	[52]
	3.2.1.55	α-L-Arabinofuranosidase	*-Enterococcus casseliflavus* *Enterococcus mundtii* *Klebsiella pneumoniae* *-* *Clostridium termitidi* *-* *Thermobacillus xylanolyticus* *-* *Caldicellulosiruptor obsidiansis*	Corn stover - α-cellulose - Filter paper - Crystalline cellulose	Exohydrolyze α-L-1,5 and/or α-L-1,3 linkages of arabinofuranosyl-based oligomers	[52] - [53] - [55] - [56]
	3.2.1.51	α-L-fucosidase	*Caldicellulosiruptor obsidiansis*	Crystalline cellulose	Cleavage α-1,6-, α-1,3-, α-1,4-, and/or α-1,2 bonds in fucosylated oligosaccharides	[56]
	3.1.1.72	acetylxylan esterase	*Enterococcus casseliflavus* *Pseudobutyrivibrio xylanivorans* *-* *Clostridium termitidi*	Corn stover - α-cellulose	Remove the O-acetyl groups from the O-2 and/or O-3 positions	[52] - [53]
	3.1.1.1	Carboxylesterase	*Caldicellulosiruptor obsidiansis*	Crystalline cellulose	Hydrolyzes ester bonds, liberating alcohol and carboxylic acid	[56]
	NIA	Esterase	*Clostridium clariflavum* *Clostridium josui* *-* *Clostrodium termitidi* *-* *Pantoea ananatis* Sd-1	Corn stover - α-cellulose - Rice straw	Cleavage ester bonds	[52] - [53] - [54]
	3.5.1.41	Chitin deacetylase	*Clostridium termitidi*	Cellobiose	Hydrolyze the N-acetoamido groups of N-acetyl-β-D-glucosaminide in chitin	[53]
	3.2.1.14	Chitinase	*Clostridium termitidi*	cellobiose	Endo-hydrolyzes N-acetyl-β-D-glucosaminide β-1,4 linkages in chitin and chitodextrins.	[53]
	3.2.1.52	β-N-acetylhexosaminidase	*Pantoea ananatis* Sd-1	Rice straw	Hydrolyse the β-1,4 glycosidic bond between N-acetylglucosamine and anhydro-N-acetylmuramic acid	[54]
	NIA	Cellulosomal proteins	*Clostridium termitidi* *-* *Clostridium josui* *Clostridium cellulolyticum*	α-cellulose - Filter paper	Protein complex that achieves hydrolysis cellulose and hemicellulose	[53] - [55]
	NIA	Cellulosomal xylanase	*Clostridium cellulolyticum*	Filter paper	Hydrolyzes β-1,4 linkages in the xylan backbone	[55]

NIA: no information available.

**Table 2:** List of cellulose and lignin degrading enzymes produced by different anaerobic bacteria.

Microorganism	Substrate (Concentration)	Identified Enzymes	Number of Different Proteins ^a^	Reference
*Clostridium thermocellum* ATCC 27405	Avicel (2 g/L)	Exoglucanase Endoglucanase Xylanase Xyloglucanase Lichenase Mannanase Chitinase Endopygalactorunase Glycosyl hydrolase	3 11 3 1 1 1 1 1 9	[57]
*Clostridium thermocellum* ATCC 27405	Cellobiose (2 g/L)	Xylanase Endoglucanase Exoglucanase Xyloglucanase Chitinase α-l-arabinofuranosidase B Glycoside hydrolase	5 9 3 1 1 2 9	[57]
*Clostridium cellulovorans*	Cellobiose (3 g/L)	Endoglucanase Mannanase Exocellulase	5 4 1	[58]
*Clostridium cellulovorans*	Avicel (3 g/L)	Endoglucanase Mannanase Xylanase Exocellulase	6 4 1 1	[58]
*Clostridium cellulovorans*	Xylan (3 g/L)	Endoglucanase Mannanase Xylanase Exocellulase	8 4 2 1	[58]
*Clostridium cellulolyticum* H10	Washed hatched wheat straw (5 g/L)	Endoglucanase Acetyl xylan esterase Mannanase Rhamnogalacturonan lyase Xylanase Cellobiohydrolase Cellulase Feruloyl esterase Xyloglucanase Arabinosidase α-arabinofuranosidase α-galactosidase β-galactosidase	17 2 2 1 10 3 1 2 1 1 1 2 1	[59]
*Pandoraea* sp. ISTKB ^*^	Kraft lignin (2 g/L)	Peroxidases Laccase Oxidases Oxidoreductases Vanillate-O-demethylase Dioxygenases Oxygenases Monooxygenase	4 1 10 16 2 13 2 1	[60]
*Aspergillus fumigatus* G-13 ^*^	p-coumaric acid (0.1 mmol/L), sinapic acid (0.1 mmol/L), glucose (10 g/L) and cellulose (10 g/L)	Dioxygenase Glyoxylase Oxidoreductase Ferulic acid esterase Monooxygenase Catalase peroxidase Cellulase β-glucancellobiohydrolase Cellobiose dehydrogenase Peroxidase Methyltransferase Oxidase Ketoreductase Aldo keto reductase Catalase	8 1 5 2 8 1 1 1 1 1 2 1 1 1 2	[61]
*Phanerochaete chrysosporium* ^*^	Softwood (30 g with 75% moisture content)	β-Glucosidase Mannanase Endoglucanase Exocellobiohydrolase Mannosidase Oxidase Lignin peroxidase	3 1 2 3 1 1 1	[62]

^a^ Number of identified proteins showing corresponding enzyme activity. ^*^ Aerobic microorganisms.

**Table 3:** Enzymes involved in different pathways of lignin degradation.

Reaction/Pathway	Enzyme	Microorganism	*Gene*	Reference
β-O-4 aryl ether	Cα-dehydrogenase	*Sphingobium sp. SYK -6*	*ligD* *ligL* *ligN* *ligO*	[68,69]
	β-etherase	*Sphingobium sp. SYK -6*	*ligF; ligE* *ligP*	[68,69]
	Glutathione lyase	*Sphingobium sp. SYK -6*	*ligG*	[68]
O-demethylation	Syringate-O-demethylase	*Sphingobium sp. SYK -6*	*desA*	[70]
	Vanillate O-demethylase	*Sphingobium sp. SYK -6*	*ligM*	[70]
Benzoyl-CoA pathway	Ligase	*Rhodopseudomonas palustris*	*hbaA*	[71]
	Reductase	*Thauera aromatica*	NIA	[72]
	pHB-CoA reductase	*Rhodopseudomonas palustris*	*hbaBCD*	[71]
	Benzoyl-CoA reductase class 1	*Thauera aromatica*	*bcrA* *bcrD* *bcrB* *bcrC*	[73]
	Benzoyl-CoA reductase class 2	*Geobacter metallireducens*	*bamB* *bamC* *bamDE* *bamCF* *bamGHI*	[74,75]
	Cyclohexadienoyl-CoA hydratase	*Geobacter metallireducens*	*bamR*	[74]
	Hydroxyenoyl-CoA dehydrogenase	*Geobacter metallireducens*	*bamQ*	[74]
	oxoacyll-CoA hydrolase	*Geobacter metallireducens*	*bamA*	[74]
β-oxidation- Benzoyl-CoA pathway	Hydroxyacyl-CoA dehydrogenase	*Geobacter metallireducens*	*pimE*	[75]
	Acyl-CoA acetyltransferase (β-Ketothiolase)	*Geobacter metallireducens*	*pimB*	[75]
	Glutaryl-CoA dehydrogenase	*Geobacter metallireducens*	*gcdH*	[75]
	3-hydroxybutyryl-CoA dehydratase	*Geobacter metallireducens*	NIA	[75]
	3-Hydroyibutyryl-CoA dehydrogenase	*Geobacter metallireducens*	NIA	[75]
	Acetoacetyl-CoA thiolase	*Geobacter metallireducens*	NIA	[75]
β-Ketoadipate pathway	Protocatechuate 3,4-dioxygenase	*Pseudomonas putida*	*pcaGH*	[76]
	Cycloisomerase	*Pseudomonas putida*	*pcaB*	[76]
	γ-Carboxy-muconolactone decarboxylase	*Pseudomonas putida*	*pcaC*	[76]
	β-ketoadipate enol-lactone hydrolase	*Pseudomonas putida*	*pacD*	[76]
	β-ketoadipate succinyl-CoA transferase	*Pseudomonas putida*	*pcaIJ*	[76]
	β-ketoadipate-CoA thiolase	*Pseudomonas putida*	*pcaF*	[76]
Phloroglucinol pathway	Gallate decarboxylase	*Lactobacillus plantarum*	*lpdB* *lpdC* *lpdD*	[77]
	Pyrogallol transhydroxylase	*Pelobacter acidigallici*	*athL* *bthL*	[78]
	Phloroglucinol reductase	*Pelobacter acidigallici*	NIA	[79]
	Dihydrophloroglucinol hydrolase	*Pelobacter acidigallici*	NIA	[79]
β-oxidation- Phloroglucinol pathway	3-hydroxyacyl-CoA dehydrogenase	*Pelobacter acidigallici*	NIA	[79]
	Acetyl CoA transferase	*Pelobacter acidigallici*	NIA	[79]
	Triacetic acid β-ketothiolase	*Pelobacter acidigallici*	NIA	[79]
	Acetoacetyl-CoA β-ketothiolase	*Pelobacter acidigallici*	NIA	[79]
	Phosphotransacetylase	*Pelobacter acidigallici*	NIA	[79]
	Acetate kinase	*Pelobacter acidigallici*	NIA	[79]

NIA: no information available.

**Table 4:** Reports on biofuels production using lignocellulosic biomass as feedstock.

Lignocellulosic Feedstock	Feedstock Preparation	Biofuel	Inoculum	Fermentation Method	Biofuel Yield	Biofuel Titer	Reference
Rice straw	Alkaline pretreatment and enzymatic hydrolysis	Biobutanol	*Clostridium acetobutylicum ATCC 824*	PVA-immobilized	0.23 g/g glucose	13.8 g/L	[88]
Sugarcane bagasse	Alkaline pretreatment and enzymatic hydrolysis	Biobutanol	*Clostridium acetobutylicum ATCC 824*	Suspended cell	0.16 g/g glucose	8.4 g/L	[88]
Rice straw	Alkaline and acid pretreatments and enzymatic hydrolysis	Biobutanol	*Clostridium beijerinckii* F-6	ABE	0.13 g/g	4.22 g/L	[89]
Rice straw	Mechanic, thermal, and acid pretreatment	Biobutanol	*Clostridium acetobutylicum NCIM 2337*	Batch	0.34 g/g	13.5 g/L	[90]
Sugarcane bagasse	Acid pretreatment and enzymatic hydrolysis	Ethanol	*Saccharomyces cerevisiae* XUSAE57	NIA	0.49 g/g	NIA	[91]
Oat spelt	Enzymatic hydrolysis	Ethanol	*Debaryomyces hansenii*	Immobilized	0.46 g/g	8.38 g/L	[92]
Wheat bran	Enzymatic hydrolysis	Ethanol	*Debaryomyces hansenii*	Immobilized	0.44 g/g	6.89 g/L	[92]
Sugarcane bagasse	Alkaline pretreatment and enzymatic hydrolysis	Ethanol	*Dekkera bruxellensis* GDB248	Anaerobic fermentation	0.42 g/g	4.5 g/g	[93]
Sweet sorghum bagasse	Alkaline pretreatment and enzymatic hydrolysis	Ethanol	*Dekkera bruxellensis* GDB248	Anaerobic fermentation	0.44 g/g	4.85 g/g	[93]
Bagasse, rice straw, corncob	Acid pretreatment	Biogas	Granular anaerobic sludge from chemical plant	Continuous anaerobic digestion	0.381 L/g COD (69.6 % CH_4_)	NIA	[94]
*A. tequilana* bagasse	Acid pretreatment	Methane	Granular anaerobic sludge from full-scale reactor	Batch anaerobic digestion	0.26 L CH_4_/g COD	NIA	[95]
Cornstalks fermentation effluents	Alkaline pretreatment	Methane	Anaerobic sludge	Batch	0.178 L CH_4_/g cornstalks	NIA	[96]
Cornstalks	Alkaline pretreatment	Hydrogen	*Clostridium thermocellum* 7072	Two-stage batch fermentation	0.074 L/g cornstalks	NIA	[96]
Cornstalks	Acid pretreatment	Hydrogen	Microbial consortium form cow dung compost	Batch	0.149 L H_2_/g TVS	NIA	[97]
Wheat straw	Acid pretreatment	Hydrogen	Microbial consortium form cow dung compost	Batch	0.068 L H_2_ g TVS	NIA	[98]

NIA: no information available. ABE: acetone, butanol, ethanol. PVA: polyvinyl alcohol. COD: chemical oxygen demand.

**Table 5:** Key enzymes up- and downregulated under different growth conditions in central carbon metabolism, pyruvate metabolism and ethanol production.

Microorganism	Conditions	Central Carbon Metabolism	Pyruvate Metabolism	Ethanol Production	References
*Clostridium cellulovorans*	Avicel	Upregulated	Upregulated	Upregulated	[22]
		ATP-dependent 6-phosphofructokinase (Clocel_2901 ^*^)	Pyruvate phosphate dikinase (Clocel_1454 ^**^, Clocel_4349 ^**^) Phosphoenolpyruvate carboxylase (Clocel_1149 ^**^)	Alcohol dehydrogenase (Clocel_3817 ^***^)	
		Downregulated	Downregulated	NIA	
		Glyceraldehyde-3-phosphate dehydrogenase (Clocel_0719 ^*^)	Malic enzyme (Clocel_0393 ^**^)		
	Glucose	Upregulated	Upregulated	Upregulated	[22]
		Glyceraldehyde-3-phosphate dehydrogenase (Clocel_0719 ^*^)	Phosphoenolpyruvate carboxylase (Clocel_1149 ^**^)	Pyruvate formate lyase (Clocel_1811 ^***^, Clocel_1812 ^***^)	
*Ethanoligenens harbinense* (YUAN-3)	Ethanol stress 50 mM	NIA	NIA	Ethanologenesis Upregulated Enzymes	[102]
				Acetaldehyde-CoA/alcohol dehydrogenase (ADU26923 ^***^)	
	Ethanol stress 100 mM	Upregulated	NIA	Ethanologenesis Upregulated Enzymes	[102]
				Acetaldehyde-CoA/alcohol dehydrogenase (ADU26923 ^***^)	
		Phosphoglycerate kinase (ADU27083 ^*^) Triosephosphate isomerase (ADU27084 ^*^) Glyceraldehyde-3-phosphate dehydrogenase (ADU28097 ^*^) 2,3-diphosphoglycerate-dependent phosphoglycerate mutase (ADU26920 ^*^) 2,3-bisphosphoglycerate-independent phosphoglycerate mutase (ADU27085 ^*^)		Ethanol tolerance Upregulated Enzymes	
				Desulfoferrodoxin (ADU28196 ^***^) Glutathione peroxidase (ADU28264 ^***^)	
	Ethanol stress 200 mM	Downregulated	NIA	Upregulated	[102]
		Carbon storage regulator protein (CsrA) (ADU28042 ^*^)		Acetaldehyde-CoA/alcohol dehydrogenase (ADE, ADU26923 ^***^)	
				Ethanol tolerance Upregulated Enzymes	
				Desulfoferrodoxin (ADU28196 ^***^)	
	Acetic acid stress	NIA	NIA	Upregulated	[103]
				Thioredoxin (ADU25713 ^***^, ADU26185 ^***^) Peroxiredoxin (ADU25886 ^***^) Alkyl hydroperoxide reductase (AhpC) subunit (ADU26936 ^***^) Glyceraldehyde-3-phosphate dehydrogenase (ADU27040 ^***^)	
*Clostridium acetobutylicum* (ATCC 824)	Cellobiose + Lignin	Upregulated in Stationary Phase	NIA	Downregulated in Stationary Phase	[104]
		2-keto-3-deoxy-6-phosphogluconate aldolase (CA_C2973 ^*^)		Acetaldehyde dehydrogenase (CA_C0162 ^***^) Aldehyde/alcohol dehydrogenase (AdhE2 ^***^)	
*Caldicellulosiruptor bescii* (DSM 6725)	C5 substrates (xylose and xylan)	Upregulated in xylan	NIA	NIA	[105]
		Extracellular solute binding proteins (ESBP) (Athe_0849 ^*^) (Athe_0089 ^*^)			
		Upregulated in xylose and xylan			
		ESBPs (Athe_0523 ^*^) (Athe_2091 ^*^) (Athe_2574 ^*^) (Athe_0847 ^*^)			
	C6 substrates (glucose, cellobiose and avicel)	Upregulated in avicel	NIA	NIA	[105]
		Glycoside hydrolases (Athe_0459 ^*^) (Athe_0460 ^*^)			
		Upregulated in glucose, cellobiose and avicel			
		Xylose isomerase (Athe_0345 ^*^) ABC transporter-related proteins (Athe_1109 ^*^) (Athe_0106 ^*^)			

NIA: no information available. ^*^ Central carbon metabolism; ^**^ pyruvate metabolism; ^***^ ethanol production.

## Data Availability

Not applicable.
